# Violence against girls during COVID-19 pandemic and associated factors in Gondar city, North West Ethiopia

**DOI:** 10.1017/S0950268822000140

**Published:** 2022-01-27

**Authors:** Ayenew Kassie, Simegnew Handebo, Asmamaw Adugna, Kegne Shitu

**Affiliations:** 1 Department of Health Education and Behavioral Sciences, Institute of Public Health, College of Medicine and Health Sciences, University of Gondar, Gondar, Ethiopia; 2 School of Public Health, St. Paul's Hospital, Millennium Medical College, Addis Ababa, Ethiopia

**Keywords:** COVID-19, Ethiopia, girls, violence

## Abstract

In Ethiopia, the magnitude of violence against girls during COVID-19 in the study area is not known. Therefore, this study aimed to assess the violence and associated factors during COVID-19 pandemic among Gondar city secondary school girls in North West Ethiopia. An institution-based cross-sectional study was conducted from January to February 2021. Data were collected from four public and two private Gondar city secondary schools. Investigators used stratified simple random sampling to select participants and the investigators used roster of the students at selected schools. Investigators collected the data using self-reported history of experiencing violence (victimisation). Investigators analysed data using descriptive statistics and multivariable logistic regression. Investigators invited a total of 371 sampled female students to complete self-administered questionnaires. The proportion of girls who experienced violence was 42.05% and psychological violence was the highest form of violence. Having a father who attended informal education (AOR = 1.95, 95% CI 1.08–3.51), ever use of social media 1.65 (AOR = 1.65, 95% CI 1.02–2.69), ever watching sexually explicit material (AOR = 2.04, 95% CI 1.24–3.36) and use of a substance (AOR = 1.92, 95% CI 1.17–3.15) were significantly associated variables with violence. Almost for every five girls, more than two of them experienced violence during the COVID-19 lockdown. The prevalence of violence might be under reported due to desirability bias. Therefore, it is better to create awareness towards violence among substance users, fathers with informal education and social media including user females.

## Background

Violence in childhood and adulthood is a universal phenomenon, usually perpetrated by people with whom children and young people interact every day in home, school and community settings [1]. Violence against children includes all forms of violence against people under 18 years old, whether perpetrated by parents or other caregivers, peers, romantic partners or strangers [2]. Up to 1 billion children aged 2–17 years have been exposed to physical, sexual, or emotional violence or neglect in the previous year, of these Africa, Asia and North America accounting for half of it [3].

COVID-19 is a significant public health emergency that has affected millions of people. It affected the already vulnerable children (twin public health) so that it is a twin public health problem [4]. Home is not a safe space for many women and girls around the world, and quarantines and lockdowns during the pandemic have increased the risk of gender-based violence. Violence is spreading like an opportunistic infection, taking advantage of the pandemic's conditions [5, 6]. Even before the pandemic, one out of every three women and girls would experience physical and/or sexual violence at some point in their lives, the majority of which will be perpetrated by an intimate partner [7].

A study carried out on children to assess the brutality against kids during the COVID-19 period indicated the prevalence form and consequence of child violence highly raised after the pandemic of COVID-19 in the world [8, 9]. The widespread reporting of rising domestic violence instances is likely to continue throughout the epidemic, and it may only be the ‘tip of the iceberg’, as many victims remain imprisoned with their abusers and unwilling to disclose the abuse [10].

Rates of drug and alcohol usage and misuse may potentially influence the risk and consequences of intimate partner violence (IPV) during the COVID-19 pandemic. Quarantine and other social isolating settings have been linked to alcohol addiction, and posttraumatic stress symptoms [13].

In Ethiopia the severity and magnitude of any form of child violence during pandemic is unacceptably increasing. Physical violence has been experienced by 23% of women aged 15–49 since they were 15, and 15% have experienced it in the last 12 months [11]. Similarly, over one-third of ever-married women had suffered some form of abuse, indicating that a significant percentage of women in the country are still victims of violence [12]. In Ethiopia, where there is an epidemic, IPV against women is linked to a girl's age, her parents' educational level, their work and media exposure [14, 15]. Although plenty of studies were carried out on violence, the magnitude of it during COVID-19 pandemic is not studied. Furthermore, the previous studies did not show the association between social media use, watching sexually explicit material, substance use and violence experiencing. This study gave insight into public health policy designers, researchers and programmes that emphasise on violence against girls to design tailored intervention. Therefore, the goal of this study was to determine the extent of violence and associated factors among young schoolgirls in Northwest Ethiopia during the COVID-19 pandemic.

## Methods

### Study design and setting

In Gondar city, an institution-based cross-sectional study was carried out from January to February 2021. Gondar city is located 727 km north of Addis Ababa in the middle Gondar zone of the Amhara regional state. According to the Gondar city health office 2017/2018 report, there are 20 administrative kebeles (the smallest administrative unit in Ethiopia) with a total population of 338 746 (160 522) males and 178 223 females, and 78 772 homes. There are 17 public and private secondary schools (grades 9–12) in the city. There were about 23 200 students from those schools, of those 12 225 were young female students [16].

### Study population, sample size and sampling technique

All female students in Gondar city were the source population and students in selected schools during data collection period were the study population. Investigators calculated the sample size based on the assumption that 50% of the respondents had experienced violence since the start of the COVID-19 pandemic. The 50% prevalence was arbitrary, as no previous study has been conducted among the study population. Investigators also assumed 95% level of confidence and 5% margin of error, and made the calculation using the following formula:
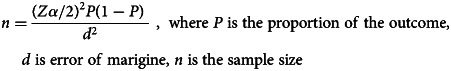

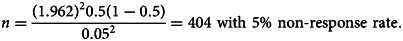


Investigators used stratified simple random sampling technique to select the study participants. First, investigators obtained the list of all private and public schools in Gondar, and randomly selected four public and two private secondary schools. Investigators then requested a roster of the students at selected schools, and randomly selected female students using Microsoft excel random number generator.

### Variables measurements

#### Violence

It is the outcome variable. During the COVID-19 epidemic, a girl who encountered at least one form of violence (psychological violence or physical violence, or sexual violence) was considered to have experienced violence. A total of 17 questions, nine for psychological violence, four for physical violence and four sexual violence, were used to measure violence. For example, psychological questions were asked like ‘During the COVID-19 lock down, have you ever been belittled or humiliated in front of other people?’, ‘During the COVID-19 lock down, have you ever been scared or intimidated of someone on purpose?’ The responses were ‘Yes’ or ‘No’. Some of physical violence questions were asked as ‘During the COVID-19 lock down, have you ever been kicked with fist or with something else that could hurt you?’, ‘During the COVID-19 lock down, have you ever been attacked with a gun, knife, or other weapons?’, ‘During the COVID-19 lock down, have you ever been scalded or burnt purposefully during the stay home period?’ and ‘During the COVID-19 lock down, have you ever been slapped, pushed, shoved, or pulled by someone?’. The responses were ‘Yes’ or ‘No’. And some of the sexual violence questions were ‘Since you have been in the school lock down period, has someone kissed or sexually touched you without your active, ongoing voluntary agreement?’, ‘During COVID-19 lock down period, has someone had contact with you involving penetration or oral sex without your active, ongoing voluntary agreement?’. The responses were ‘Yes’ or ‘No’.

#### Substance use

An individual who uses at least one substance (alcohol, khat or cigarette) during COVID-19 pandemic was considered as substance user (Yes = 1) and those who did not use any of the substance were considered as non-users (No = 0) [15].

#### Use of social media

A student who ever uses at least one type of social media (Facebook, Telegram, Twitter or Instagram) during COVID-19 pandemic was considered as they use social media (Yes = 1) while those who did not use any of them were considered as they didn't use (No = 0).

### Data collection procedure

A one-day training was given to both the data collectors and supervisors by the principal investigators about the purpose of the study, data collection procedures and ethical issues during data collection. The selected female students were requested for written consent to participate in the study and for those aged below 18, their parents also requested for written consent. They were interviewed individually and the interview place was in the private place. Their partners were not participating in the interview to keep confidentiality and allow the female students to freely explain the experience of violence and to minimise conflict with their partners. If any member of the family was nearby, the interview time was shifted to another period.

### Study instrument development and validation

Data were collected by a pretested self-administered structured questionnaire. After analysing several literatures, the investigator created the questionnaire [12, 14–17]. There were three sections to the questionnaire. The first segment dealt with the participants' socio-demographic factors, the second with substance abuse, and the third with violence-related variables. The investigators initially constructed the questionnaire in English, and then forward and backward translation was done by both Amharic and English-speaking personnel to ensure consistency. On the basis of the translators' translation reports, necessary adjustments to the instrument were made.

Six experts from the health behaviour, infectious disease and COVID-19 pandemic response teams participated in a content validity test. Item level Content Validity Index (I-CVI) of 0.78 or higher, Scale level Content Validity Index by Universal Agreement (S-CVI/UA) of 0.8 or higher, and Scale level Content Validity Index by Average (S-CVI/Ave) of 0.9 or higher were used to assess it. In addition, the instrument's reliability was assessed using Cronbach *α*'s internal consistency reliability assessment.

### Data analysis

EPI DATA version 4.6.2 was used to clean and code the data, which was then exported to STATA version 14 for analysis. Frequencies and proportions were computed as descriptive statistics. The statistical significance was determined using a 95% confidence level. Binary logistic regression was used to detect the association of secondary school student violence. To control for potential confounders and determine the significance of the association, a multivariable logistic regression analysis was utilised. Furthermore, the size of the relationship between different independent factors and the dependent variable was calculated using odd ratios with a 95% confidence interval. The Hosmor–Lemeshow goodness of fit test was performed to assess the model's suitability.

## Results

### Socio-demographic characteristics

Of 404 recruited participants, 371 students were participated giving a response rate of 92%. The table below shows demographic distribution of the study participants. More than one-third of the participants were grade 12^th^ students and half of the participant's age was less than 18 years old (Table 1).

**Table 1.** Gondar city female secondary school students' socio-demographic features in 2021, North West Ethiopia (*n* = 371)

Variable	Category	Frequency	Per cent
Age	<18 years old	188	50.67
	≥18years old	183	49.33
Educational status	9th	29	7.82
	10th	98	26.42
	11th	110	29.65
	12th	134	36.12
Marital status	Unmarried	332	89.49
	Engaged/married	39	10.51
Religion	Orthodox Christian	329	88.68
	Muslim	28	7.55
	Others	14	3.77
Mother's education	Informal educational status	152	40.97
	Attend primary school	53	14.29
	Attend secondary school	68	18.33
	Attend diploma and above	98	26.42
Father's education	Informal educational status	140	37.74
	Attend primary school	46	12.40
	Attend secondary school	80	21.56
	Diploma and above	105	28.30
Mother's occupation	Farmer/housewife	234	63.07
	Government employee	77	20.75
	Merchant	42	11.32
	Others	18	4.85
Father's occupation	Employee	136	36.66
	Farmer	96	25.88
	Merchant	95	25.61
	Others	44	11.86

### Experience of violence

**Fig. 1.**
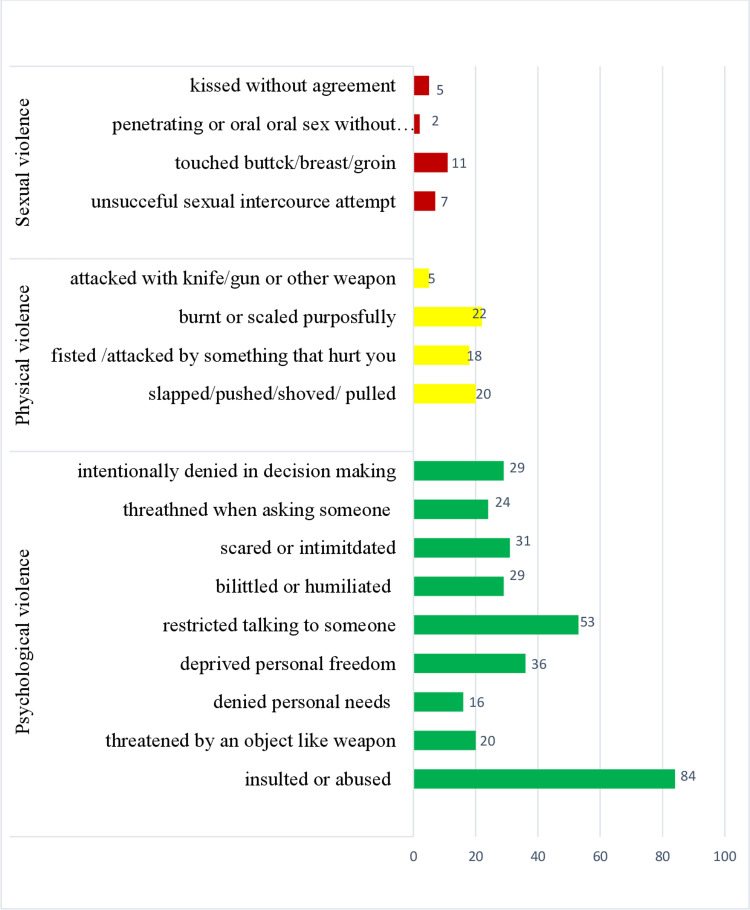
Types of violence of Gondar city secondary school female students, North West Ethiopia, 2021.

The experience of all types (either emotional or physical or sexual) of violence during lock down due to COVID-19 was 42.05% (95% CI 37.1–47.16%). Psychological violence was by far the most common sort of violence (39.5%), with 22.6% reported being insulted by someone during a COVID-19 lockdown and 7.82% reported being humiliated in front of others. The second form of violence was physical violence, about 11.59% of the female students experienced physical violence during lock down due to COVID-19. About 6% experienced burn purposefully on their body. Sexual violence was the least common kind of violence during the COVID-19 lockdown. About 4.04% of Gondar city secondary school female students reported that they had experienced sexual violence during lock down due to COVID-19. The participants were asked the perpetrators and four of them, 36 and three of those who experienced psychological violence, physical violence and sexual violence reported that the perpetrators were relatives (Fig. 1).

### Utilisation of social media, mass and substance use media during COVID-19 lockdown

During COVID-19 lockdown, social media was used by more than half of the participants (56.87%), of which 58.77%, 30.81% and 6.64% used Facebook, Telegram and Instagram, respectively. About 46.63% and 14.56% watched television and listened to radio daily respectively. Almost one-fourth (25.07%) of female students watched sexually explicit material during COVID-19 lockdown. About 14.28% of the students used substances such as alcohol, khat and cigarette.

### Associated factors of violence against female students

We, investigators, used bivariable and multivariable logistic regression to find the associated factors of violence in Gondar during the COVID-19 lockdown. Father's educational status, social media use, watching television, watching sexually explicit material, substance use, seeing their mother being insulted were variables with a *P*-value of <0.2. On multivariable logistic regression, father's educational status, ever use of social media, watching sexually explicit material and substance use were statistically significant variables which had association with violence of secondary school students during COVID-19 lock down.

The odds of violence during COVID-19 lock down among students whose father's educational status of informal were 1.95 (AOR = 1.95; CI 1.08–3.51) times higher than those whose father's educational status was diploma and above. Those participants who ever used social media were 1.65 (AOR = 1.65; CI 1.02–2.92) times more likely to experience violence during COVID-19 lock down than who did not use. The odds of violence during COVID-19 lock down among participants who ever watched sexually explicit materials were 2.04 (AOR = 2.04; CI 1.24–3.36) times more likely than those who did not watched. Substance use was the contributing factor for violence. Those participants who currently use substance (alcohol, khat and cigarette) were 1.92 (AOR = 1.92; CI 1.17–3.15) times more likely to experience violence during COVID-19 lock down (Table 2).

**Table 2.** Multivariable logistic regression of experience of violence among Gondar city secondary students during COVID-19 lock down, North West Ethiopia, 2021 (*n* = 371)

Variable		Experience violence			
		Yes (%)	No (%)	AOR (95% CI)	*P*-value
Father's education	Diploma and above	35 (33.33)	70 (66.67)	1	1
	Secondary education	33 (41.25)	47 (58.75)	1.31 (0.71–2.46)	0.39
	Primary education	75 (53.57)	23 (50.00)	2.03 (0.97–4.28)	0.06
	Informal education	65 (46.43)	75 (53.57**)**	1.95 (1.08–3.51)	0.02*
Used social media in life time	No	61 (38.13)	99 (61.88)	1	1
	Yes	95 (45.02)	116 (54.98)	1.65 (1.02–2.69)	0.04*
Frequency of watching television	Daily	70 (40.46)	103 (40.46)	1	1
	Sometimes	50 (40.32)	74 (59.68)	1.06 (0.65–1.74)	0.81
	Never	36 (48.65)	38 (51.35)	1.36 (0.72–2.59)	0.33
History of watching sexually explicit materials	No	103 (37.05)	175 (62.95)	1	1
	Yes	53 (56.99)	40 (43.01)	2.04 (1.24–3.36)	<0.01*
History of substance use	No	103 (37.45)	172 (62.55)	1	1
	Yes	53 (55.21)	43 (44.79)	1.92 (1.17–3.15)	<0.01*
History of witnessing verbal violence against mother	No	131 (39.82)	198 (60.18)	1	1
	Yes	25 (59.52)	17 (40.48)	0.57 (0.28–1.16)	0.12

## Discussion

The goal of the study was to determine the factors of violence among female secondary school students in Gondar, North West Ethiopia, during the COVID-19 lockdown. Having father who had informal education, ever use of social media, watching sexual explicit material and substance use were associated variables which had association with violence during COVID-19 lock down.

This study showed that the experience of violence during COVID-19 lock down among Gondar city female students was 42.05%. Psychological violence was the most prevalent, and this is also confirmed with different literatures [18, 19]. This result is higher than those researches conducted in Northern Ethiopia [15] and Paris [20]. The discrepancy could be related to demographic differences (a study in Paris). According to studies, violence is linked to a person's or a community's level of education and economic standing [21]. Paris is with higher socio-economic status than Ethiopia. It might be due to this reason that the prevalence of violence in Paris is lower. The other possible justification for the variation of our study and a study in Northern Ethiopia might be that our study assessed all types of violence by anyone (the perpetrator could be intimate partner, parents, relatives, neighbour, unknown individuals) but the Northern Ethiopia study only assessed IPV. So that the prevalence could be lower than ours. The experience of violence was found to be lower in this study than in a Brazilian study [10]. This could be that drug use is common among Brazilian youths so that they might commit violence against girls [23].

This study revealed that father's level of education was associated with violence. Students having a father who had informal education level were more likely to experience violence. This result is consistent with researches done in Ethiopia [12, 22]. In our study, most of the perpetrators of emotional and physical violence were parents like mother and father. Educated fathers are less likely to commit violence compared to illiterates [23], and those illiterate parents might believe biting and insulting is a way of parenting style to shape their children.

Our study showed that the use of social media such as Facebook, Telegram and Twitter was associated with violence. Those students who ever use social media were more likely to be subjected to violence. This finding is also revealed in other studies [24]. This could be that frequent social media users might gap the relationship with their families because they spent most of their time with social media, so that parents might physically or emotionally violate against them.

The finding of this study demonstrated that substance use during COVID-19 lock down, such as khat, alcohol and cigarettes, was a significant factor of violence. This is consistent with studies conducted in different countries [9, 21–23]. Students who use substance like khat, alcohol and cigarette are more likely to lose their consciousness/in difficulty to decide what should and shouldn't so that they might be vulnerable to violence. Students who use substance had the chance of high contact with other substance abusers and this increases their vulnerability to violence [25]. The study found that watching sexual explicit material was a significant associated factor of violence. Studies conducted in different literatures support the finding [26–28]. Watching sexual materials like pornography provoke individuals to sexual behaviour [29–31].

### Limitation of the study

Although this research showed the magnitude and factors of violence victims during COV-19 pandemic, it has also some caveats. Since violence is sensitive, there might be social desirability bias and this might lower the percentage of victims who experienced violence. Furthermore, this study did not report the intensity, frequency and duration of violence.

## Conclusion

Almost for every five girls, more than two of them experienced violence during COVID-19 lock down. Having informal educational level of father educational status, ever use of social media, watching sexually explicit material and substance use were significant determinants of violence during COVID-19 lock down. Awareness creation intervention about violence is needed on fathers who attended informal educational level, substance users and watch sexually explicit material. Health education should be delivered for female secondary school students on the negative consequence of watching sexually explicit material and substance use. Furthermore, public health programmes who worked on violence better consider provision of intervention on the utilisation of social media, association of substance use and violence.

## Data Availability

The datasets created and/or analysed in this study are available from the principal investigator upon reasonable request (Ayenew Kassie, E-mail kassieayenew@gmail.com).
